# Effect of water intake on routine laboratory test parameters in healthy adults: a cross-sectional study

**DOI:** 10.3389/fmed.2026.1884690

**Published:** 2026-07-08

**Authors:** Zhuang-Zhuang Zhai, Xue-Jing Yu, Ru-Yue Chen, Xue-Yan Lv, Yue-Hang Wang, Li Bai, Li Li, Qing-Lei Zeng

**Affiliations:** Department of Infectious Diseases and Hepatology, The First Affiliated Hospital of Zhengzhou University, Zhengzhou, Henan, China

**Keywords:** biological variation, clinical chemistry tests, complete blood count, reference values, water intake

## Abstract

**Introduction:**

Fasting before routine blood sampling has been regarded as a standard requirement. However, this practice may cause unnecessary discomfort and lacks sufficient scientific evidence. This study aimed to investigate the effect of water intake on commonly measured complete blood count (CBC) and biochemical parameters.

**Materials and methods:**

This two-step study involved two homogeneous cohorts of healthy adult medical postgraduate students. In Step 1, participants from Cohort 1 (*n* = 38) underwent standard fasting conditions to establish the short-term biological variation (BV) and corresponding reference change values (RCVs). In Step 2, participants from Cohort 2 (*n* = 33) had blood analysis before and one hour after consuming 500 mL water to assess the resulting changes, and only mean differences (MDs) exceeding the corresponding RCVs were considered clinically significant.

**Results:**

Twelve CBC parameters and 7 biochemical parameters showed statistically significant differences one hour after the intake of 500 mL of water compared with pre-intake values, including leukocytes, lymphocytes, monocytes, erythrocytes, hemoglobin, hematocrit, mean cell volume, mean cell hemoglobin, mean cell hemoglobin concentration, red cell distribution width–standard deviation, platelets, plateletcrit, alkaline phosphatase, total cholesterol, high-density lipoprotein cholesterol, low-density lipoprotein cholesterol, urea, uric acid, and creatinine. The remaining 6 CBC parameters and 5 biochemical parameters showed no statistically significant differences, including neutrophils, eosinophils, basophils, mean platelet volume, platelet distribution width, platelet larger cell ratio, alanine aminotransferase, aspartate aminotransferase, *γ*-glutamyltransferase, triglyceride, and glucose. Notably, only the change of lymphocytes was considered clinically significant.

**Conclusion:**

Water intake before routine blood sampling has minimal clinically relevant effects on commonly measured CBC and biochemical parameters in healthy adults.

## 1 Introduction

Among all factors affecting laboratory test results, the pre-analytical phase is the most error-prone (1, 2). A major source of pre-analytical variation arises from patients’ inadequate understanding of pre-test requirements (3). The related hydration status is believed to affect the accuracy of blood test results (4). Consequently, strict fasting before blood sampling has long been regarded as a standard requirement, often including restriction of water intake (5). In clinical practice, withholding water can cause unnecessary discomfort, especially for individuals accustomed to drinking water first thing in the morning. Current consensus suggests that water intake does not affect lipid profiles (6), and a recent study indicates that even fasting duration can be appropriately shortened without compromising the reliability of laboratory results (7). However, high-quality evidence regarding its effects on other commonly measured laboratory analytes remains limited.

When investigating whether short-term interventions, such as water intake, induce clinically significant changes in blood analytes, the reference change value (RCV) serves as an ideal metric. Unlike statistical significance, RCV is specifically designed to identify changes that are meaningful in a clinical context and is widely accepted in clinical practice (8–10). Calculation of RCV depends on biological variation (BV) data (9–11). The European Federation of Clinical Chemistry and Laboratory Medicine (EFLM) has established standardized procedures for evaluating high-quality BV data and has compiled a database that currently represents the most comprehensive BV resource worldwide (12, 13). However, most data in the EFLM database reflect only inter-day BV, and there is a notable scarcity of intra-day BV data, which are essential for calculating RCVs to assess the effects of short-term interventions like water intake.

In this study, we first followed the EFLM’s standardized procedures to evaluate intra-day, short-term BV at 30-min intervals for commonly measured analytes and derived the corresponding RCVs as thresholds for distinguishing clinically significant changes (Step 1). Subsequently, we assessed whether water intake could induce clinically significant changes in these analytes (Step 2), addressing the scientific question of whether drinking water affects routine blood test results.

## 2 Materials and methods

This study was conducted at The First Affiliated Hospital of Zhengzhou University, China. The study protocol was designed and performed in strict accordance with the ethical principles outlined in the Declaration of Helsinki. This study was approved by the Ethics Committee for Scientific Research and Clinical Trials of the First Affiliated Hospital of Zhengzhou University. Separate approvals were obtained for the two distinct Steps of this study: Step 1 (No. 2024-KY-1610-002) and Step 2 (No. 2025-KY-1338-001). Furthermore, the trials were prospectively registered with the Chinese Clinical Trial Registry (Step 1: ChiCTR2400093261; Step 2: ChiCTR2500113064).

### 2.1 Subjects

In both Step 1 and Step 2, participants were healthy volunteer medical postgraduate students from Zhengzhou University. Step 1 and Step 2 each involved a separate cohort, namely Cohort 1 and Cohort 2. All participants completed a screening questionnaire based on the inclusion and exclusion criteria, and their eligibility was confirmed by the investigation team.

Participants were eligible if they provided written informed consent, reported good mental and physical health, were older than 18 years, were not currently taking any medications (including over-the-counter drugs or supplements), and had no history of smoking or alcohol consumption.

Participants were excluded if they had diabetes requiring medication or fasting plasma glucose >7.0 mmol/L, chronic liver or kidney disease, dyslipidemia (total cholesterol >6.5 mmol/L), a family history of thalassemia or other hemoglobinopathies, severe chronic or acute illnesses (e.g., cancer, cardiovascular, or neurological disorders), were carriers of hepatitis B, hepatitis C, or HIV, had been hospitalized or seriously ill in the preceding 4 weeks, had donated blood within the past 3 months, were pregnant, breastfeeding, or within 1 year postpartum, or had other conditions deemed unsuitable by investigators (e.g., history of syncope during venipuncture, hemophilia).

### 2.2 Procedure

#### 2.2.1 Step 1 (Cohort 1)

Participants were instructed to begin strict fasting after 19:00 on the day prior to blood sampling and to refrain from strenuous exercise or emotional agitation. On the day of blood sampling, they arrived at the collection site between 07:40 and 07:50 and remained seated for at least 5 min before the first blood draw.

An indwelling intravenous cannula was inserted into the median cubital vein prior to the first blood draw. Venous blood samples were then collected via the cannula at 08:00 and subsequently at 30-min intervals, resulting in a total of five collections per participant.

Blood samples were immediately transported to the laboratory for processing after collection. To minimize inter-batch variation, all specimens were analyzed within a single analytical run, with duplicate measurements performed for each analyte (38 participants×5 time points×2 replicates).

#### 2.2.2 Step 2 (Cohort 2)

Participant preparation was identical to that in Step 1. To minimize procedural differences between the two steps, an indwelling intravenous cannula was also used for blood collection following the same protocol. After the first blood draw via the cannula at 08:00 (T1), participants were instructed to consume 500 mL of water within 5 min. A volume of 500 mL was selected because it represents the approximate upper limit of habitual morning water consumption in East Asia and some other regions, while also matching the capacity of a standard commercially available water bottle. Following a one-hour seated rest at the sampling site, the second blood draw (T2) was performed. Physical activity, including walking and positional changes beyond normal seated adjustments, was not permitted during this time. Blood storage and transport procedures were the same as in Step 1. However, blood samples collected at T1 (before water intake) and T2 (1 hour after water intake) were analyzed together in a single run (33 participants×2 time points).

### 2.3 Laboratory analysis

This study measured a total of 30 commonly assessed analytes, including 18 complete blood count (CBC) parameters and 12 biochemical parameters. The 18 CBC parameters were: leukocytes, neutrophils, eosinophils, basophils, lymphocytes, monocytes, erythrocytes, hemoglobin (Hb), hematocrit (Hct), mean cell volume (MCV), mean cell hemoglobin (MCH), mean cell hemoglobin concentration (MCHC), red cell distribution width–standard deviation (RDW-SD), platelets (Plt), mean platelet volume (MPV), plateletcrit, platelet distribution width (PDW), and platelet larger cell ratio (P-LCR). The 12 biochemical parameters were: alanine aminotransferase (ALT), aspartate aminotransferase (AST), *γ*-glutamyltransferase (GGT), alkaline phosphatase (ALP), total cholesterol (TC), triglycerides (TG), high-density lipoprotein cholesterol (HDL), low-density lipoprotein cholesterol (LDL), uric acid (UA), urea, creatinine (CREA), and glucose (Glc).

The water consumed by participants was bottled water (Want-Want Group, Taipei, Taiwan, China) that had been treated by reverse osmosis, filtration, and high-temperature sterilization to ensure its physical, chemical, and biological quality. Blood samples were collected using sterile, safety-engineered, closed-system intravenous indwelling needles paired with evacuated blood collection tubes (Ande Medical Corp., Zibo, Shandong, China). Hematological and biochemical parameters were analyzed using a Sysmex XN-3000 hematology analyzer (Sysmex Corp. Kobe, Chuo Ward, Japan) and a Beckman AU5800 automated biochemical analyzer (Beckman Coulter Diagnostics, Brea, California, United States), respectively, with the corresponding reagents and calibrators. Analytical methods for the CBC and biochemical parameters are summarized in Supplementary Tables 1, 2.

### 2.4 Statistical analysis

The estimates of analytical variation (CV_A_) and within-subject biological variation (CV_I_) were calculated using the CV-ANOVA (14), a robust method for variance component analysis (15). Measurement data were first normalized and expressed as coefficients of variation for nested analysis of variance to determine CV_A_ and CV_I_. Before nested analysis, outliers were assessed and removed. Homogeneity of variances was tested using the Bartlett test for CV_A_ and the Cochran test for CV_I_. To confirm that participants were in a steady state, linear regression was performed on the mean concentration across the 5 sampling points for each parameter. If the 95% confidence interval (CI) of the regression slope included zero, it was considered to be in a steady state. The Shapiro–Wilk test was applied to verify the normality of the residuals. If the normal distribution assumption was rejected, the natural logarithmic scale transformation was used. The 95% CI was calculated according to Sahai’s method (14), and statistical significance was defined as the lack of overlap between the 95% CIs.

RCV was calculated from the BV estimates (8, 9, 16). For RCV calculation, Z_𝛼_ was set at 1.64 (*α* = 0.05). For each participant, the measurement values before water intake and after 1 hour were recorded as T_1_ and T_2_ for each parameter, respectively. The mean difference (MD) represented the mean percentage of change for each parameter (17, 18). If MD exceeded the corresponding RCV, it was considered a clinically significant change. The results of the paired statistical comparison were also presented. If the difference between T_1_ and T_2_ followed a normal distribution, the paired *t*-test was used; otherwise, the Wilcoxon signed-rank test was used.

Exploratory stratified subgroup analyses were conducted by sex. And continuous linear regression of individual MD on estimated blood volume (Nadler formula) was also performed to evaluate whether body size influenced the dilutional effect. Bonferroni correction was applied to all subgroup comparisons and regression models for 30 simultaneous tests. All equations were as follows:


$$SD_{A,\log}^{2}=\log_{e}(CV_{A}^{2}+1)$$

$$SD_{I,\log}^{2}=\log_{e}(CV_{I}^{2}+1)$$

$$\begin{array}{c} SD^{*}=\sqrt{SD_{A,\log}^{2}+SD_{I,\log}^{2}} \end{array}$$

$$\begin{array}{c} \mathrm{RCV}\%=100\%\times e^{\left(\pm Z_{\alpha }\times \sqrt{2}\times SD^{*}\right)}-1 \end{array}$$

$$\begin{array}{c} \mathrm{MD}\%=\frac{{\bar{T}}_{2}-{\bar{T}}_{1}}{{\bar{T}}_{1}}\times 100\% \end{array}$$

Blood Volume(male)=0.3669×Height+0.03219×Weight+0.6041

$$\begin{array}{l} \text{Blood Volume}\;(\text{female})=0.3561\times \text{Height}+0.03308\times \\ \text{Weight}+0.1833 \end{array}$$

All statistical analyses were performed using IBM SPSS Statistics version 26.0 (IBM Corp., Chicago, IL, United States). For statistical description, if the data followed the normal distribution, mean (standard deviation (SD)) was used to represent; otherwise, median (interquartile range (IQR)) was used to represent.

## 3 Results

### 3.1 Subjects characteristics

In Step 1, a total of 38 participants (16 males and 22 females) were enrolled, with a mean age of 24.53 ± 1.18 years and a mean body mass index (BMI) of 21.92 ± 2.45 kg/m^2^. In Step 2, 33 participants (20 males and 13 females) were enrolled, with a mean age of 24.53 (SD 1.18) years and a mean BMI of 23.05 (SD 3.13) kg/m^2^. All participants had abstained from medications or vitamin supplementation for at least 1 month prior to the study and reported no history of smoking or alcohol consumption. Detailed demographic characteristics are presented in Table 1.

**Table 1:** Detailed demographic characteristics of participants in Step 1 (Cohort 1) and Step 2 (Cohort 2).

Characteristics	Step 1 (Cohort 1)	Step 2 (Cohort 2)	χ^2^/*t*/*Z*	*p* value
*n*	38	33	NA	NA
Mean (SD) age, years	24.53 (1.18)	24.39 (1.25)	0.44	0.660
Mean (SD) height, m	1.67 (0.09)	1.71 (0.09)	1.59	0.120
Mean (SD) weight, kg	61.55 (9.67)	67.61 (12.89)	1.35	0.180
Mean (SD) BMI, kg/m^2^	21.92 (2.45)	23.05 (3.13)	0.60	0.550
No (%) male sex	16 (42.11)	20 (60.61)	2.42	0.120

NA, not applicable; χ^2^, Chi-Square value for Chi-Square test; *t*, *t*-value for *t*-test; *Z*, *Z*-value for Wilcoxon rank-sum test; BMI, body mass index; SD, standard deviation.

### 3.2 Biological variation estimates in Step 1

For each parameter, a total of 380 values were initially measured (38 participants×5 time points×2 replicates). The outcomes of outlier exclusion are summarized in Supplementary Table 3. Except for HDL, the proportion of excluded values was less than 10% for all parameters. All included datasets met the normality assumption, and no significant temporal trends were observed for any parameter over the course of the study.

The CV_A_ and CV_I_ for each parameter, along with their 95% CIs, are presented in Table 2. The CV_I_ estimates ranged from approximately zero (basophils, MCV, MCHC, plateletcrit and ALP) to 20.47% (eosinophils), whereas CV_A_ estimates ranged from 0.67% (MCV) to 37.61% (basophils).

**Table 2:** Step 1 (Cohort 1): biological variation estimates for hematological and biochemical parameters.

Parameter	This study			EFLM database	
	Mean (95%CI)	CV_I_ (95%CI), %	CV_A_ (95%CI), %	CV_I_ (95%CI), %	Update time
Leukocytes (10^9^/L)	5.71 (5.58, 5.84)	5.8 (5.2, 6.6)	2.6 (2.3, 2.9)	11.1 (8.2, 12.8)	February 11, 2026
Neutrophils (10^9^/L)	3.36 (3.26, 3.47)	6.6 (6.0, 7.4)	3.7 (3.3, 4.1)	12.5 (6.3, 26.9)	February 11, 2026
Eosinophils (10^9^/L)	0.05 (0.04, 0.06)	20.5 (18.3, 23.3)	19.4 (17.5, 21.7)	15.1 (10.1, 26.9)	January 21, 2026
Basophils (10^9^/L)	0.02 (0.01, 0.02)	≈0 (≈0, ≈0)	37.6 (33.7, 42.6)	12.6 (11.3, 32.0)	January 21, 2026
Lymphocytes (10^9^/L)	1.94 (1.89, 1.99)	8.7 (7.8, 9.8)	3.9 (3.6, 4.4)	10.5 (9.8, 14.7)	February 11, 2026
Monocytes (10^9^/L)	0.29 (0.28, 0.30)	8.4 (7.5, 9.4)	8.1 (7.4, 9.0)	14.0 (11.0, 19.3)	February 11, 2026
Erythrocytes (10^12^/L)	4.47 (4.43, 4.51)	2.0 (1.8, 2.3)	2.9 (2.6, 3.2)	2.6 (1.4, 4.0)	February 11, 2026
Hb (g/L)	132.74 (131.01, 134.46)	1.0 (0.9, 1.1)	4.1 (3.8, 4.6)	2.7 (1.7, 2.7)	February 11, 2026
Hct (%)	40.53 (40.06, 41.00)	2.1 (1.9, 2.4)	2.7 (2.5, 3.0)	2.8 (2.2, 3.1)	February 11, 2026
MCV (fl)	90.95 (90.62, 91.29)	≈0 (≈0, ≈0)	0.7 (0.6, 0.8)	0.8 (0.6, 1.6)	February 11, 2026
MCH (pg)	30.07 (29.93, 30.20)	0.2 (0.2, 0.3)	0.8 (0.7, 0.9)	0.7 (0.3, 1.5)	February 11, 2026
MCHC (g/L)	329.58 (328.75, 330.40)	≈0 (≈0, ≈0)	1.0 (0.9, 1.1)	1.0 (0.5, 1.8)	February 11, 2026
RDW-SD (fl)	42.12 (41.93, 42.31)	0.2 (0.1, 0.2)	0.9 (0.8, 1.0)	1.7 (1.4, 3.7)	February 11, 2026
Plt (10^9^/L)	241.07 (236.27, 245.86)	1.7 (1.5, 1.9)	6.2 (5.6, 7.0)	7.3 (6.6, 10.2)	February 11, 2026
MPV (fl)	10.25 (10.14, 10.36)	1.6 (1.5, 1.8)	1.5 (1.3, 1.6)	2.3 (2.2, 4.3)	February 20, 2025
Plateletcrit (%)	0.26 (0.25, 0.27)	≈0 (≈0, ≈0)	5.9 (5.3, 6.6)	6.4 (6.4, 11.6)	February 20, 2025
PDW (%)	16.10 (16.06, 16.13)	0.4 (0.4, 0.5)	0.8 (0.7, 0.9)	3.8 (3.2, 4.2)	February 20, 2025
P-LCR (%)	27.98 (27.18, 28.79)	4.1 (3.7, 4.6)	4.3 (3.9, 4.5)	6.8 (6.6, 7.0)	February 20, 2025
ALT (U/L)	10.51 (10.08, 10.94)	3.6 (3.2, 4.0)	11.9 (10.8, 13.3)	12.6 (9.3, 17.3)	August 13, 2025
AST (U/L)	18.49 (18.02, 18.97)	7.2 (6.6, 8.4)	8.0 (7.2, 8.9)	8.6 (6.2, 13.8)	August 13, 2025
GGT (U/L)	12.29 (11.95, 12.63)	5.1 (4.5, 5.8)	6.4 (5.7, 7.3)	8.3 (6.7, 13.4)	August 13, 2025
ALP (U/L)	57.42 (56.16, 58.68)	≈0 (≈0, 0.02)	6.9 (6.3, 7.7)	5.0 (2.6, 5.5)	October 21, 2025
TC (mmol/L)	4.56 (4.48, 4.63)	1.7 (1.6, 2.0)	1.0 (0.9, 1.1)	5.3 (4.9, 6.3)	February 25, 2026
TG (mmol/L)	0.71 (0.68, 0.74)	5.5 (4.9, 6.3)	2.3 (2.1, 2.6)	19.8 (17.9, 21.9)	February 25, 2026
HDL (mmol/L)	1.30 (1.28, 1.32)	1.9 (1.7, 2.1)	1.8 (1.6, 2.0)	5.7 (4.7, 7.7)	February 25, 2026
LDL (mmol/L)	2.44 (2.38, 2.49)	1.8 (1.6, 2.0)	1.8 (1.6, 2.0)	7.8 (7.0, 8.4)	February 25, 2026
Urea (mmol/L)	4.09 (4.00, 4.17)	2.3 (2.0, 2.6)	1.5 (1.4, 1.7)	13.1 (11.2, 14.1)	January 21, 2026
UA (μmol/L)	263.70 (255.91, 271.49)	1.4 (1.2, 1.6)	0.9 (0.8, 1.0)	8.3 (5.5, 9.8)	January 21, 2026
CREA (μmol/L)	57.11 (56.05, 58.18)	2.9 (2.6, 3.3)	1.7 (1.6, 1.9)	4.4 (2.3, 5.0)	February 20, 2026
Glc (mmol/L)	3.09 (3.05, 3.13)	7.4 (6.6, 8.4)	2.0 (1.8, 2.2)	4.7 (3.0, 5.4)	February 23, 2026

CV_I_, within subject biological variation; CV_A_, analytical variation; EFLM, European Federation of Clinical Chemistry and Laboratory Medicine; CI, confidence interval; Hb, hemoglobin; Hct, hematocrit; MCV, mean cell volume; MCH, mean cell hemoglobin; MCHC, mean cell hemoglobin concentration; RDW-SD, red cell distribution width–standard deviation; Plt, platelets; MPV, mean platelet volume; PDW, platelet distribution width; P-LCR, Platelet larger cell ratio; ALT, alanine aminotransferase; AST, aspartate aminotransferase; GGT, γ-glutamyltransferase; ALP, alkaline phosphatase; TC, total cholesterol; TG, triglyceride; HDL, high-density lipoprotein cholesterol; LDL, low-density lipoprotein cholesterol; UA, uric acid; CREA, creatinine; Glc, glucose.

### 3.3 Paired statistical comparison and comparison between MD with RCV

Normality test results for the differences between T1 and T2 are presented in Supplementary Table 4. Following water intake, paired statistical comparisons indicated significant changes in several parameters: leukocytes (*t* = −6.16, *p* < 0.001), lymphocytes (*t* = −10.83, *p* < 0.001), monocytes (*t* = −2.80, *p* = 0.009), erythrocytes (*T* = 88.5, *p* = 0.001), Hb (*T* = 54.00, *p* = 0.001), Hct (*T* = 143.00, *p* = 0.041), MCV (*t* = 5.02, *p* < 0.001), MCH (*t* = −2.84, *p* = 0.008), MCHC (*t* = −4.99, *p* < 0.001), RDW-SD (*t* = 3.68, *p* = 0.001), Plt (*t* = −6.23, *p* < 0.001), plateletcrit (*T* = 12.00, *p* < 0.001), ALP (*T* = 333.00, *p* = 0.039), TC (*T* = 78.50, *p* < 0.001), HDL (*T* = 79.50, *p* = 0.001), LDL (*t* = −4.42, *p* < 0.001), urea (*t* = −4.69, *p* < 0.001), UA (*t* = −4.56, *p* < 0.001), and CREA (*t* = −5.17, *p* < 0.001). However, only lymphocytes showed a clinically significant change, with a MD of −20.5% relative to the RCV of −19.8 to 24.6%. Complete details are provided in Tables 3, 4.

**Table 3:** Step 2 (Cohort 2): paired statistical comparison between T_1_ and T_2_.

Parameter	${\bar{T}}_{1}$ (95%CI)	${\bar{T}}_{2}$ (95%CI)	Difference [mean (SD)/median (IQR)]	*t*/*T*	*P*
Leukocytes (10^9^/L)	5.99 (5.46, 6.52)	5.43 (4.92, 5.94)	−0.56 (0.53)	−6.16	<0.001
Neutrophils (10^9^/L)	3.10 (2.75, 3.44)	3.06 (2.70, 3.41)	−0.12 (−0.27, 0.12)	192.5	0.118
Eosinophils (10^9^/L)	0.14 (0.11, 0.18)	0.14 (0.10, 0.18)	−0.01 (−0.02, 0.01)	165	0.572
Basophils (10^9^/L)	0.03 (0.02, 0.03)	0.02 (0.02, 0.03)	≈0 (−0.01, ≈0)	72	0.077
Lymphocytes (10^9^/L)	2.37 (2.16, 2.57)	1.87 (1.70, 2.05)	−0.49 (0.26)	−10.83	<0.001
Monocytes (10^9^/L)	0.36 (0.32, 0.40)	0.33 (0.29, 0.37)	−0.03 (0.06)	−2.80	0.009
Erythrocytes (10^12^/L)	4.76 (4.59, 4.93)	4.70 (4.54, 4.87)	−0.07 (−0.11, −0.01)	88.5	0.001
Hb (g/L)	143.97 (139.06, 148.88)	141.45 (136.70, 146.21)	−3.00 (−5.00, −1.00)	54	0.001
Hct (%)	42.48 (41.03, 43.93)	42.18 (40.75, 43.61)	−0.40 (−0.80, 0.10)	143	0.041
MCV (fl)	89.25 (88.38, 90.12)	89.71 (88.82, 90.59)	0.45 (0.52)	5.02	<0.001
MCH (pg)	30.24 (29.92, 30.56)	30.10 (29.80, 30.40)	−0.14 (0.28)	−2.84	0.008
MCHC (g/L)	338.91 (336.75, 341.07)	335.45 (333.62, 337.29)	−3.45 (3.97)	−4.99	<0.001
RDW-SD (fl)	41.21 (40.51, 41.92)	41.54 (40.80, 42.28)	0.33 (0.52)	3.68	0.001
Plt (10^9^/L)	261.82 (241.19, 282.45)	250.94 (230.86, 271.02)	−10.88 (10.03)	−6.23	<0.001
MPV (fl)	9.80 (9.52, 10.08)	9.74 (9.49, 9.99)	0 (−0.20, 0.10)	159.5	0.485
Plateletcrit (%)	0.25 (0.24, 0.27)	0.24 (0.22, 0.26)	−0.01 (−0.02, 0)	12	<0.001
PDW (%)	16.12 (16.02, 16.23)	16.08 (15.96, 16.20)	−0.04 (0.21)	−1.16	0.256
P-LCR (%)	24.64 (22.76, 26.51)	24.16 (22.39, 25.94)	−0.47 (1.96)	−1.38	0.176
ALT (U/L)	23.61 (18.11, 29.10)	23.97 (18.71, 29.23)	0.36 (2.47)	0.85	0.405
AST (U/L)	21.24 (18.39, 24.09)	21.30 (18.58, 24.02)	0.06 (2.46)	0.14	0.888
GGT (U/L)	22.36 (16.88, 27.85)	22.03 (16.73, 27.33)	0 (−1.00, 0)	97	0.117
ALP (U/L)	53.79 (47.74, 59.84)	59.67 (52.96, 66.37)	2.00 (−2.00, 18.00)	333	0.039
TC (mmol/L)	4.52 (4.24, 4.80)	4.42 (4.15, 4.69)	−0.11 (−0.22, −0.04)	78.5	<0.001
TG (mmol/L)	1.07 (0.90, 1.24)	1.09 (0.90, 1.28)	0.02 (0.11)	0.84	0.405
HDL (mmol/L)	1.27 (1.19, 1.35)	1.25 (1.17, 1.33)	−0.03 (−0.05, −0.01)	79.5	0.001
LDL (mmol/L)	2.64 (2.41, 2.87)	2.56 (2.34, 2.78)	−0.08 (0.10)	−4.42	<0.001
Urea (mmol/L)	4.56 (4.16, 4.96)	4.45 (4.06, 4.85)	−0.11 (0.13)	−4.69	<0.001
UA (μmol/L)	337.94 (300.63, 375.25)	334.00 (296.74, 371.26)	−3.94 (4.96)	−4.56	<0.001
CREA (μmol/L)	73.12 (68.04, 78.20)	71.06 (66.11, 76.01)	−2.06 (2.29)	−5.17	<0.001
Glc (mmol/L)	4.33 (4.17, 4.49)	4.41 (4.28, 4.54)	0.08 (0.24)	1.96	0.059

${\bar{T}}_{1}$ , mean of participant parameters measurement values before water intake; ${\bar{T}}_{2}$ , mean of participant parameters measurement values 1 hour after drinking 500 mL of water; CI, confidence interval; SD, standard deviation; IQR, interquartile range; *t*, *t*-value for paired *t*-test; *T*, *T*-value for Wilcoxon signed-rank test; Hb, hemoglobin; Hct, hematocrit; MCV, mean cell volume; MCH, mean cell hemoglobin; MCHC, mean cell hemoglobin concentration; RDW-SD, red cell distribution width–standard deviation; Plt, platelets; MPV, mean platelet volume; PDW, platelet distribution width; P-LCR, Platelet larger cell ratio; ALT, alanine aminotransferase; AST, aspartate aminotransferase; GGT, γ-glutamyltransferase; ALP, alkaline phosphatase; TC, total cholesterol; TG, triglyceride; HDL, high-density lipoprotein cholesterol; LDL, low-density lipoprotein cholesterol; UA, uric acid; CREA, creatinine; Glc, glucose.

**Table 4:** Step 2 (Cohort 2): comparison of mean difference with reference change value.

Parameter	RCV (95%CI), %		MD (%)
	This study	EFLM BV database	
Leukocytes (10^9^/L)	(−13.7, 15.9)	(−23.2, 30.3)	−9.51
Neutrophils (10^9^/L)	(−16.1, 19.2)	(−26.1, 35.4)	−1.43
Eosinophils (10^9^/L)	(−47.7, 91.1)	(−43.3, 76.2)	−5.25
Basophils (10^9^/L)	(−57.0, 132.5)	(−59.1, 144.8)	−8.23
Lymphocytes (10^9^/L)	(−19.8, 24.6)	(−22.9, 29.8)	−20.54
Monocytes (10^9^/L)	(−23.6, 30.9)	(−31.2, 45.4)	−8.08
Erythrocytes (10^12^/L)	(−7.8, 8.5)	(−8.6, 9.4)	−1.21
Hb (g/L)	(−9.4, 10.4)	(−10.8, 12.1)	−1.70
Hct (%)	(−7.7, 8.4)	(−8.7, 9.5)	−0.67
MCV (fl)	(−1.6, 1.6)	(−2.4, 2.5)	0.51
MCH (pg)	(−1.9, 1.9)	(−2.5, 2.5)	−0.45
MCHC (g/L)	(−2.4, 2.4)	(−3.2, 3.4)	−1.01
RDW-SD (fl)	(−2.0, 2.1)	(−4.4, 4.5)	0.80
Plt (10^9^/L)	(−13.8, 16.1)	(−19.9, 24.9)	−4.15
MPV (fl)	(−4.9, 5.2)	(−6.1, 6.5)	−0.48
Plateletcrit (%)	(−12.7, 14.6)	(−18.3, 22.4)	−4.71
PDW (%)	(−2.1, 2.1)	(−8.7, 9.6)	−0.26
P-LCR (%)	(−12.9, 14.8)	(−17.0, 20.5)	−1.36
ALT (U/L)	(−25.0, 33.2)	(−33.1, 49.5)	4.80
AST (U/L)	(−21.9, 28.1)	(−23.5, 31.2)	1.18
GGT (U/L)	(−17.2, 20.8)	(−21.6, 27.6)	−0.98
ALP (U/L)	(−14.8, 17.4)	(−18.0, 22.0)	13.05
TC (mmol/L)	(−4.6, 4.8)	(−11.8, 13.4)	−2.24
TG (mmol/L)	(−12.9, 14.8)	(−36.9, 58.4)	0.60
HDL (mmol/L)	(−5.8, 6.2)	(−13.0, 15.0)	−1.88
LDL (mmol/L)	(−5.6, 5.9)	(−17.0, 20.4)	−2.87
Urea (mmol/L)	(−6.1, 6.5)	(−26.4, 35.8)	−2.31
UA (μmol/L)	(−3.8, 3.9)	(−17.5, 21.3)	−1.27
CREA (μmol/L)	(−7.5, 8.2)	(−10.4, 11.6)	−2.78
Glc (mmol/L)	(−16.3, 19.4)	(−11.1, 12.5)	2.26

RCV, reference change value; EFLM, European Federation of Clinical Chemistry and Laboratory Medicine; BV, biological variation; MD, mean difference; Hb, hemoglobin; Hct, hematocrit; MCV, mean cell volume; MCH, mean cell hemoglobin; MCHC, mean cell hemoglobin concentration; RDW-SD, red cell distribution width–standard deviation; Plt, platelets; MPV, mean platelet volume; PDW, platelet distribution width; P-LCR, Platelet larger cell ratio; ALT, alanine aminotransferase; AST, aspartate aminotransferase; GGT, γ-glutamyltransferase; ALP, alkaline phosphatase; TC, total cholesterol; TG, triglyceride; HDL, high-density lipoprotein cholesterol; LDL, low-density lipoprotein cholesterol; UA, uric acid; CREA, creatinine; Glc, glucose.

### 3.4 Exploratory subgroup analyses

In sex-stratified subgroup analysis, after Bonferroni correction for 30 comparisons, no parameter remained significant (Supplementary Table 5). Linear regression of individual MD on estimated blood volume was performed for each parameter (Supplementary Table 6), and no parameter showed a regression slope significantly different from zero after Bonferroni correction.

## 4 Discussion

### 4.1 Principal findings

**Figure 1:**
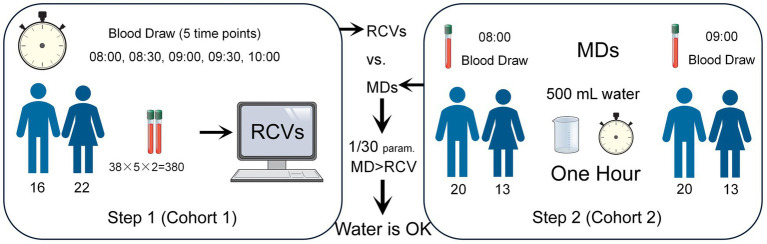
Study design and findings. RCVs for 30 parameters were established in Cohort 1 (*n* = 38) based on blood samples collected at five time points, with duplicate measurements performed (38 × 5 × 2 = 380). In Cohort 2 (*n* = 33), MDs were calculated by comparing blood samples obtained before and 1 hour after the consumption of 500 mL of water. Only one parameter exhibited a MD that exceeded its corresponding RCV. MD, mean difference; param., parameter(s); RCV, reference change value.

This study established short-term CV_I_ and RCVs at 30-min intervals for 18 CBC parameters and 12 biochemical parameters, and demonstrated that intake of 500 mL of water resulted in clinically significant changes only in lymphocyte counts among the analytes examined (Figure 1). These findings indicate that water intake has a negligible impact on routine blood test results in healthy young adults, providing important evidence to inform pre-analytical fasting requirements in health examination populations worldwide.

### 4.2 Compared with other studies

Our results build upon and differ from those reported in previous studies. Although recent guidelines permit habitual water intake, the absence of specific quantitative recommendations has resulted in a lack of uniform standards for medical practitioners in routine clinical practice (5, 19). Consequently, patients are often instructed to undergo complete fasting, including both food and liquids (20), which may lead to unnecessary discomfort. Benozzi et al. conducted two analogous studies and reported that intake of 300 mL of water did not result in clinically significant changes in commonly measured CBC and biochemical parameters (17, 18). Notably, however, the CV_I_ and corresponding RCVs applied in those studies were derived from the Westgard database (last updated in 2014), and participant homogeneity was not rigorously controlled, potentially limiting comparability (21). Therefore, a rigorously designed study is warranted to more accurately evaluate the effects of water intake on laboratory test results and to provide robust evidence to guide clinical practice. Recently, Solé-Enrech et al. evaluated the impact of reducing fasting hours on laboratory results in a Mediterranean population and demonstrated that a fasting period of 4–6 h is sufficient for reliable results across commonly measured analytes, further supporting the notion that current fasting requirements may be unnecessarily stringent (7).

### 4.3 Rigorous evaluation

We recruited healthy young medical postgraduate students from Zhengzhou University according to the inclusion and exclusion criteria in Step 1 and Step 2, resulting in a high specimen utilization rate, good compliance, homogeneous cohorts, and representative specimens from healthy young adults. In Step 1, a total of 38 individuals were included, with 5 venous blood specimens collected from each individual over a 2-h morning period, meeting the recommendations regarding the number of subjects and specimens proposed by Braga and Panteghini (22). The number of individuals for Step 2 was planned to be the same as that in Step 1. However, only 33 individuals were recruited for Step 2. Therefore, a post-hoc sensitivity analysis was conducted, which confirmed that the reduced sample still maintained ≥80% power to detect clinically meaningful changes for all parameters (Supplementary Table 7). To minimize the discomfort of repeated venipuncture over the short period, this study employed indwelling catheters for repeated blood sampling. Numerous studies have demonstrated that this method does not affect the quality of blood specimens (23–26). Blood sampling in this study was conducted in the morning (08:00–10:00), consistent with the practices of routine blood sampling time and Step 2 (27, 28).

In Step 1, the reliable short-term RCV was calculated based on BV data. CV_I_ represents the variations of analytes around the homeostatic set point within individuals (29). Estimating BV values for relevant analytes is of great importance in laboratory medicine and clinical practice (13, 15). The EFLM initiated the European Biological Variation Study in 2014 and published quality assessment criteria for BV studies, which have profoundly advanced laboratory medicine (8, 12). However, the current EFLM BV database lacks studies on short-term (within hours) BV, forcing some researchers to rely on long-term BV data when evaluating short-term (e.g., within 2 h) intervention effects, leading to unreliable conclusions (17, 18). Following the quality assessment checklist provided by EFLM, this short-term BV study on common CBC and biochemical parameters found that for most parameters, both the estimated CV_I_ values and RCVs were reduced compared to those in the EFLM BV database.

The estimated CV_I_ values in this study differed from those reported in the EFLM BV database. Except for eosinophil and Glc, the CV_I_ estimates of the remaining 28 parameters were lower than the values reported in the EFLM BV database. Furthermore, with the exception of neutrophils, eosinophils, erythrocytes, Hct, AST, and CREA, the remaining 24 parameters’ CV_I_ estimates exhibited statistically significant differences compared to the results from the EFLM BV database. The CV_I_ estimates of basophils, MCV, MCHC, plateletcrit, and ALP were approximately zero. These differences may be related to several factors. First, they may be associated with the sampling intervals used in the study design. The EFLM BV database, compiled by EFLM through meta-analyses of existing studies, mostly includes studies with sampling intervals of 1 week or longer (30, 31), whereas the sampling interval in this study was 0.5 h with a total observation duration of 2 h. Objectively, longer sampling intervals between blood specimen collections would increase the likelihood of confounding factors affecting homeostasis (32). Additionally, the differences may relate to the characteristics of the recruited populations, as few BV studies based on Chinese young populations are included in the EFLM BV database, while this study targeted Chinese healthy young individuals. The CV_I_ estimates for neutrophils, eosinophils, erythrocytes, Hct, AST, and CREA did not show statistically significant differences compared with the EFLM BV database reports, indicating that CV_I_ of these parameters may be insensitive to sampling interval and remains relatively stable across populations (33).

Similar to CV_I_, RCVs for the 28 parameters in this study (excluding eosinophils and Glc) were narrower than the corresponding RCVs calculated based on the EFLM BV database. Since the value of RCV is directly related to the estimated CV_I_, this similarity may also be attributed to the short interval between specimen collections and the characteristics of the populations. RCV is a longitudinal indicator used to monitor change in an individual’s test results, representing the threshold at which consecutive measurement results exhibit clinically significant change (9). Notably, compared to other parameters in this study, the RCV ranges for eosinophils (−47.7 to 91.1%) and basophils (−57.0 to 132.5%) were the widest, indicating that the short-term physiological fluctuations of eosinophils and basophils within individual homeostasis remain substantial.

The paired statistical comparison results showed that the changes of 12 CBC parameters (leukocytes, lymphocytes, monocytes, erythrocytes, Hb, Hct, MCV, MCH, MCHC, RDW-SD, Plt, and plateletcrit) and 7 biochemical parameters (ALP, TC, HDL, LDL, urea, UA, and CREA) among these commonly measured analytes were statistically significant (Table 3). However, in Benozzi et al.’s studies (17, 18), 4 CBC parameters (MCV, MCH, RDW-SD, and Plt) and 3 biochemical parameters (HDL, LDL, and CREA) among these parameters did not show statistically significant changes. The difference may derive from the volume of water intake (500 mL water intake in the current study *vs.* 300 mL water intake in Benozzi et al.’s studies). The effect of drinking water on analytes was not simply a dilutional effect. Notably, some analytes showed increases in their concentrations after water intake: MCV, RDW-SD, ALT, AST, ALP, TG, and Glc. The increases of MCV, RDW-SD, and ALP were statistically significant (Table 3). This phenomenon also existed in Benozzi et al.’s studies (17, 18). This demonstrates the complex homeostatic self-regulation within the human body. Although the comparison of RCV (−19.8 to 24.6%) with MD (−20.5%) in lymphocytes indicated clinically significant changes, the decrease was also near the threshold of the RCV range. For instance, even with a 20.5% decline at the lower limit of the 95% CI, the lymphocytes in this study (from 2.16 × 10^9^/L to 1.72 × 10^9^/L) remained within the population reference range. This also suggests the strong homeostatic self-regulation within the human body.

Moreover, neither sex-stratified comparisons nor blood-volume regression identified significant dilutional differences following Bonferroni correction (Supplementary Tables 5, 6). The rapid distribution of ingested water into total body water likely buffered against individual differences in body size over this one-hour interval.

### 4.4 Strengths and limitations

This study has several notable strengths. First, a rigorous and tightly controlled study design was employed, ensuring high internal validity. Second, this study is, to our knowledge, the first to strictly follow the EFLM standardized procedures to evaluate intra-day, short-term biological variation at 30-min intervals for commonly measured analytes and to derive corresponding RCVs as thresholds for distinguishing clinically significant changes. Third, all participants were recruited from a medical school, resulting in a highly homogeneous study population with consistent organization, protocol adherence, and participant compliance. Leveraging these strengths, this study estimated short-term within-subject biological variation for 18 CBC parameters and 12 biochemical parameters in healthy young individuals and derived related metrics, providing robust data for the evaluation of short-term interventions. The results further demonstrated that intake of 500 mL of water resulted in only marginally clinically significant changes in lymphocyte counts.

Nevertheless, several limitations of this study should be acknowledged. First, different volumes of water intake were not evaluated, precluding the determination of an optimal or threshold intake level. However, given that consumption of 500 mL of water in the morning represents an upper-limit volume for most individuals undergoing health examinations, this study was designed to directly assess the effects of a potentially maximal water intake. Second, only healthy young adults were included; therefore, the generalizability of these findings to other populations, such as patients, older adults, or children, may be limited. In older adults, the age-related decline in glomerular filtration rate may delay water clearance, and impaired cardiovascular autonomic regulation may result in greater hemodynamic fluctuations following fluid intake. In pediatric populations, differences in body surface area-to-volume ratios and immature homeostatic control mechanisms may produce divergent patterns of analyte change. In patients with chronic kidney disease, impaired renal excretory capacity could amplify and extend the dilutional impact of water ingestion. These physiological distinctions underscore the need for population-specific validation studies before the current findings can be generalized beyond healthy young adults. As noted above, the study employed a highly intensive sampling protocol, with blood collection every 30 min in the Step 1 cohort, which would be ethically and practically challenging to implement in more vulnerable populations. Third, this study only assessed the effect of purified water and should not be directly extrapolated to beverages containing other solutes. Fourth, the non-significant findings of the sex-stratified comparisons and blood volume regression analyses should be interpreted with caution due to the limited sample size.

## 5 Conclusion

The findings of this study provide evidence questioning the necessity of restricting water intake before blood sampling and suggest that routine morning water consumption in customary amounts is unlikely to meaningfully affect blood test results, particularly in health examination settings involving healthy individuals. Whether these conclusions can be generalized to other populations, such as patients, older adults, or children, warrants further investigation in future studies.

## Data Availability

The raw data supporting the conclusions of this article will be made available by the authors, without undue reservation.
