# Supplementary material for: Effect of water intake on routine laboratory test parameters in healthy adults: a cross-sectional study
Source: Front Med (Lausanne). 2026 Jul 8;13:1884690. doi: 10.3389/fmed.2026.1884690 (PMC13388096; doi:10.3389/fmed.2026.1884690)
Supplement: Supplementary file 1 [file Table_1.DOCX]

**S Table 1. Analytical Methods for Hematological Parameters.**

| **Parameter** | **Method, Unit** | **Parameter** | **Method, Unit** |
| --- | --- | --- | --- |
| Leukocytes | Impedance method, ×10^9^/L | MCV | Impedance method, fl |
| Neutrophils | VCS technology, ×10^9^/L | MCH | Calculation method, pg |
| Eosinophils | VCS technology, ×10^9^/L | MCHC | Calculation method, g/L |
| Basophils | WBC/BASO technology, ×10^9^/L | RDW-SD | Impedance method, fl |
| Lymphocytes | VCS technology, ×10^9^/L | Plt | Impedance method, ×10^9^/L |
| Monocytes | VCS technology, ×10^9^/L | MPV | Impedance method, fl |
| Erythrocytes | Impedance method,×10^12^/L | Plateletcrit | Impedance method, % |
| Hb | Colorimetric method, g/L | PDW | Impedance method, % |
| Hct | Calculation method, % | P-LCR | Impedance method, % |

Hb, hemoglobin; Hct, hematocrit; MCV, mean cell volume; MCH, mean cell hemoglobin; MCHC, mean cell hemoglobin concentration; RDW-SD, red cell distribution width–standard deviation; Plt, platelet count; MPV, mean platelet volume; PDW, platelet distribution width; P-LCR, Platelet larger cell ratio.

**S Table 2. Analytical Methods for Biochemical Parameters.**

| **Parameter** | **Method, Unit** | **Parameter** | **Method, Unit** |
| --- | --- | --- | --- |
| ALT | Rate method, U/L | HDL | Direct method, mmol/L |
| AST | Rate method, U/L | LDL | Direct method, mmol/L |
| GGT | Rate method, U/L | UA | Uricase UV method, μmol/L |
| ALP | Rate method, U/L | CREA | Enzymatic method, μmol/L |
| TC | Cholesterol oxidase method, mmol/L | Urea | Urea Enzymatic UV method, mmol/L |
| TG | Enzymatic method, mmol/L | Glc | Glccose oxidase method, mmol/L |

ALT, alanine aminotransferase; AST, aspartate aminotransferase; GGT, γ-glutamyltransferase; ALP, alkaline phosphatase; TC, total cholesterol; TG, triglyceride; HDL, high-density lipoprotein cholesterol; LDL, low-density lipoprotein cholesterol; UA, uric acid; CREA, creatinine; Glc, glucose.

**S Table 3. Inclusion and Exclusion Results for Hematological Parameters and** **Biochemical Parameters.**

| **Parameter** | **Numbers of results used to estimate CV_I_ data** | |  | **Data recognized as outliers** | | |
| --- | --- | --- | --- | --- | --- | --- |
|  | **Subjects** | **Results** |  | **Excluded results** | **Excluded subjects** | **Total of outliers, %** |
| Leukocytes | 37 | 362 |  | 1 | 18 | 4.7 |
| Neutrophils | 38 | 370 |  | 0 | 10 | 2.6 |
| Eosinophils | 35 | 346 |  | 3 | 34 | 8.9 |
| Basophils | 38 | 380 |  | 0 | 0 | 0 |
| Lymphocytes | 38 | 378 |  | 0 | 2 | 0.5 |
| Monocytes | 38 | 376 |  | 0 | 4 | 1.1 |
| Erythrocytes | 35 | 348 |  | 3 | 32 | 8.4 |
| Hb | 38 | 376 |  | 0 | 4 | 1.1 |
| Hct | 37 | 368 |  | 1 | 12 | 3.2 |
| MCV | 35 | 350 |  | 3 | 30 | 7.9 |
| MCH | 35 | 348 |  | 3 | 32 | 8.4 |
| MCHC | 37 | 358 |  | 1 | 22 | 5.8 |
| RDW-SD | 38 | 378 |  | 0 | 2 | 0.5 |
| Plt | 35 | 350 |  | 3 | 30 | 7.9 |
| MPV | 38 | 374 |  | 0 | 6 | 1.6 |
| Plateletcrit | 36 | 352 |  | 2 | 24 | 6.3 |
| PDW | 38 | 380 |  | 0 | 0 | 0 |
| P-LCR | 37 | 368 |  | 1 | 12 | 3.2 |
| ALT | 35 | 345 |  | 3 | 35 | 9.2 |
| AST | 35 | 348 |  | 3 | 32 | 8.4 |
| GGT | 36 | 352 |  | 2 | 28 | 7.4 |
| ALP | 37 | 370 |  | 1 | 10 | 2.6 |
| TC | 35 | 350 |  | 3 | 30 | 7.9 |
| TG | 35 | 346 |  | 3 | 34 | 8.9 |
| HDL | 34 | 340 |  | 4 | 40 | 10.5 |
| LDL | 35 | 350 |  | 3 | 30 | 7.9 |
| UA | 38 | 378 |  | 0 | 2 | 0.5 |
| Urea | 36 | 358 |  | 2 | 22 | 5.8 |
| CREA | 37 | 368 |  | 1 | 12 | 3.2 |
| Glc | 37 | 370 |  | 1 | 10 | 2.6 |

Hb, hemoglobin; Hct, hematocrit; MCV, mean cell volume; MCH, mean cell hemoglobin; MCHC, mean cell hemoglobin concentration; RDW-SD, red cell distribution width–standard deviation; Plt, platelet count; MPV, mean platelet volume; PDW, platelet distribution width; P-LCR, Platelet larger cell ratio; ALT, alanine aminotransferase; AST, aspartate aminotransferase; GGT, γ-glutamyltransferase; ALP, alkaline phosphatase; TC, total cholesterol; TG, triglyceride; HDL, high-density lipoprotein cholesterol; LDL, low-density lipoprotein cholesterol; UA, uric acid; CREA, creatinine; Glc, glucose.

**S Table 4. Shapiro–Wilk test of the differences between T1 and T2.**

| **Parameter, unit** | **‾T_1_ (‾*x*±*s*/** **M[*P*_25_,*P*_75_])** | **‾T_2_ (‾*x*±*s*/ M[*P*_25_,*P*_75_])** | ***W*** | ***P*** |
| --- | --- | --- | --- | --- |
| Leukocytes, 10^9^/L | 5.99 (5.46, 6.52) | 5.43 (4.92, 5.94) | 0.965 | 0.358 |
| Neutrophils, 10^9^/L | 3.10 (2.75, 3.44) | 3.06 (2.70, 3.41) | 0.793 | ＜0.001 |
| Eosinophils, 10^9^/L | 0.14 (0.11, 0.18) | 0.14 (0.10, 0.18) | 0.837 | ＜0.001 |
| Basophils, 10^9^/L | 0.03 (0.02, 0.03) | 0.02 (0.02, 0.03) | 0.884 | 0.002 |
| Lymphocytes, 10^9^/L | 2.37 (2.16, 2.57) | 1.87 (1.70, 2.05) | 0.955 | 0.189 |
| Monocytes, 10^9^/L | 0.36 (0.32, 0.40) | 0.33 (0.29, 0.37) | 0.961 | 0.281 |
| Erythrocytes, 10^12^/L | 4.76 (4.59, 4.93) | 4.70 (4.54, 4.87) | 0.906 | 0.007 |
| Hb, g/L | 143.97 (139.06, 148.88) | 141.45 (136.70, 146.21) | 0.904 | 0.006 |
| Hct, % | 42.48 (41.03, 43.93) | 42.18 (40.75, 43.61) | 0.914 | 0.012 |
| MCV, fl | 89.25 (88.38, 90.12) | 89.71 (88.82, 90.59) | 0.967 | 0.403 |
| MCH, pg | 30.24 (29.92, 30.56) | 30.10 (29.80, 30.40) | 0.957 | 0.216 |
| MCHC, g/L | 338.91 (336.75, 341.07) | 335.45 (333.62, 337.29) | 0.954 | 0.177 |
| RDW-SD, fl | 41.21 (40.51, 41.92) | 41.54 (40.80, 42.28) | 0.96 | 0.26 |
| Plt, 10^9^/L | 261.82 (241.19, 282.45) | 250.94 (230.86, 271.02) | 0.969 | 0.461 |
| MPV, fl | 9.80 (9.52, 10.08) | 9.74 (9.49, 9.99) | 0.923 | 0.022 |
| Plateletcrit, % | 0.25 (0.24, 0.27) | 0.24 (0.22, 0.26) | 0.929 | 0.032 |
| PDW, % | 16.12 (16.02, 16.23) | 16.08 (15.96, 16.20) | 0.94 | 0.067 |
| P-LCR, % | 24.64 (22.76, 26.51) | 24.16 (22.39, 25.94) | 0.964 | 0.337 |
| ALT, U/L | 23.61 (18.11, 29.10) | 23.97 (18.71, 29.23) | 0.971 | 0.506 |
| AST, U/L | 21.24 (18.39, 24.09) | 21.30 (18.58, 24.02) | 0.977 | 0.704 |
| GGT, U/L | 22.36 (16.88, 27.85) | 22.03 (16.73, 27.33) | 0.890 | 0.003 |
| ALP, U/L | 53.79 (47.74, 59.84) | 59.67 (52.96, 66.37) | 0.916 | 0.014 |
| TC, mmol/L | 4.52 (4.24, 4.80) | 4.42 (4.15, 4.69) | 0.896 | 0.004 |
| TG, mmol/L | 1.07 (0.90, 1.24) | 1.09 (0.90, 1.28) | 0.965 | 0.344 |
| HDL, mmol/L | 1.27 (1.19, 1.35) | 1.25 (1.17, 1.33) | 0.821 | ＜0.001 |
| LDL, mmol/L | 2.64 (2.41, 2.87) | 2.56 (2.34, 2.78) | 0.973 | 0.563 |
| Urea, mmol/L | 4.56 (4.16, 4.96) | 4.45 (4.06, 4.85) | 0.976 | 0.648 |
| UA, μmol/L | 337.94 (300.63, 375.25) | 334.00 (296.74, 371.26) | 0.957 | 0.212 |
| CREA, μmol/L | 73.12 (68.04, 78.20) | 71.06 (66.11, 76.01) | 0.968 | 0.419 |
| Glc, mmol/L | 4.33 (4.17, 4.49) | 4.41 (4.28, 4.54) | 0.956 | 0.195 |

Hb, hemoglobin; Hct, hematocrit; MCV, mean cell volume; MCH, mean cell hemoglobin; MCHC, mean cell hemoglobin concentration; RDW-SD, red cell distribution width–standard deviation; Plt, platelet count; MPV, mean platelet volume; PDW, platelet distribution width; P-LCR, Platelet larger cell ratio; ALT, alanine aminotransferase; AST, aspartate aminotransferase; GGT, γ-glutamyltransferase; ALP, alkaline phosphatase; TC, total cholesterol; TG, triglyceride; HDL, high-density lipoprotein cholesterol; LDL, low-density lipoprotein cholesterol; UA, uric acid; CREA, creatinine; Glc, glucose.

**S Table 5. Sex-stratified Subgroup Analysis of MD After Water Intake (Bonferroni threshold *P* < 0.0017).**

| **Parameter** | **MD of Male (95%CI), %** | **MD of Female(95%CI), %** | ***P*** |
| --- | --- | --- | --- |
| Leukocytes | -7.36 (-9.96, -4.75) | -12.83 (-19.74, -5.92) | 0.1287 |
| Neutrophils | -0.02 (-3.58, 3.53) | -3.60 (-14.61, 7.42) | 0.0341 |
| Eosinophils | 0.50 (-6.77, 7.77) | -14.09 (-29.03, 0.85) | 0.0744 |
| Basophils | -0.17 (-21.52, 21.19) | -20.64 (-39.81, -1.48) | 0.1390 |
| Lymphocytes | -17.70 (-21.38, -14.02) | -24.92 (-31.05, -18.78) | 0.0417 |
| Monocytes | -2.18 (-8.13, 3.78) | -17.16 (-25.31, -9.01) | 0.0041 |
| Erythrocytes | -1.22 (-2.50, 0.05) | -1.20 (-2.60, 0.21) | 0.7823 |
| Hb | -1.77 (-2.92, -0.63) | -1.58 (-3.42, 0.25) | 0.8394 |
| Hct | -0.67 (-2.14, 0.80) | -0.66 (-2.23, 0.91) | 0.6451 |
| MCV | 0.50 (0.24, 0.75) | 0.52 (0.14, 0.91) | 0.8956 |
| MCH | -0.47 (-0.89, -0.06) | -0.42 (-1.02, 0.18) | 0.8669 |
| MCHC | -1.09 (-1.67, -0.52) | -0.88 (-1.55, -0.20) | 0.6019 |
| RDW-SD | 0.80 (0.35, 1.25) | 0.80 (-0.18, 1.77) | 0.9898 |
| Plt | -4.27 (-6.15, -2.39) | -3.95 (-6.14, -1.76) | 0.8156 |
| MPV | -0.39 (-2.01, 1.22) | -0.62 (-1.62, 0.38) | 0.8024 |
| Plateletcrit | -5.07 (-7.86, -2.28) | -4.16 (-7.18, -1.14) | 0.6384 |
| PDW | -0.31 (-1.03, 0.42) | -0.19 (-0.69, 0.31) | 0.7797 |
| P-LCR | -1.43 (-6.06, 3.20) | -1.24 (-4.20, 1.72) | 0.9430 |
| ALT | 2.86 (-4.50, 10.22) | 7.79 (-2.74, 18.32) | 0.2853 |
| AST | -1.58 (-6.48, 3.32) | 5.43 (-2.37, 13.23) | 0.1159 |
| GGT | -0.85 (-4.23, 2.53) | -1.18 (-5.16, 2.79) | 1.0000 |
| ALP | 11.88 (-0.07, 23.83) | 14.84 (2.91, 26.77) | 0.7110 |
| TC | -2.36 (-3.81, -0.91) | -2.05 (-4.62, 0.52) | 0.9266 |
| TG | 4.51 (-0.47, 9.49) | -5.40 (-10.73, -0.07) | 0.0071 |
| HDL | -2.14 (-3.83, -0.46) | -1.47 (-4.07, 1.12) | 0.7682 |
| LDL | -2.63 (-4.13, -1.13) | -3.24 (-5.96, -0.52) | 0.6763 |
| UA | -1.74 (-2.93, -0.55) | -3.18 (-5.05, -1.31) | 0.1750 |
| Urea | -0.93 (-1.61, -0.26) | -1.79 (-2.93, -0.66) | 0.1732 |
| CREA | -2.00 (-3.65, -0.35) | -3.99 (-5.39, -2.59) | 0.0604 |
| Glc | 2.91 (0.19, 5.62) | 1.26 (-2.16, 4.67) | 0.4261 |

Hb, hemoglobin; Hct, hematocrit; MCV, mean cell volume; MCH, mean cell hemoglobin; MCHC, mean cell hemoglobin concentration; RDW-SD, red cell distribution width–standard deviation; Plt, platelet count; MPV, mean platelet volume; PDW, platelet distribution width; P-LCR, Platelet larger cell ratio; ALT, alanine aminotransferase; AST, aspartate aminotransferase; GGT, γ-glutamyltransferase; ALP, alkaline phosphatase; TC, total cholesterol; TG, triglyceride; HDL, high-density lipoprotein cholesterol; LDL, low-density lipoprotein cholesterol; UA, uric acid; CREA, creatinine; Glc, glucose.

**S Table 6. Linear Regression of Individual MD on Blood Volume (Nadler formula, Bonferroni threshold *P* < 0.0017).**

| **Parameter** | **Intercept** | **Slope (95%CI)** | **R^2^** | ***P*** |
| --- | --- | --- | --- | --- |
| Leukocytes | -22.56 | 0.0029 (-0.0007, 0.0065) | 0.082 | 0.1054 |
| Neutrophils | -13.36 | 0.0027 (-0.0027, 0.0081) | 0.032 | 0.3205 |
| Eosinophils | -46.04 | 0.0092 (0.0009, 0.0174) | 0.142 | 0.0308 |
| Basophils | -56.25 | 0.0108 (-0.0067, 0.0283) | 0.049 | 0.2170 |
| Lymphocytes | -36.52 | 0.0036 (-0.0002, 0.0074) | 0.105 | 0.0659 |
| Monocytes | -44.14 | 0.0081 (0.0024, 0.0138) | 0.215 | 0.0067 |
| Erythrocytes | -1.78 | 0.0001 (-0.0010, 0.0012) | 0.002 | 0.8136 |
| Hb | -2.32 | 0.0001 (-0.0010, 0.0013) | 0.002 | 0.8062 |
| Hct | -1.51 | 0.0002 (-0.0011, 0.0014) | 0.003 | 0.7589 |
| MCV | 0.34 | 0.0000 (-0.0002, 0.0003) | 0.003 | 0.7553 |
| MCH | -0.63 | 0.0000 (-0.0004, 0.0004) | 0.001 | 0.8415 |
| MCHC | -0.77 | -0.0001 (-0.0006, 0.0005) | 0.001 | 0.8314 |
| RDW-SD | 0.40 | 0.0001 (-0.0004, 0.0006) | 0.004 | 0.7300 |
| Plt | -2.88 | -0.0003 (-0.0019, 0.0014) | 0.004 | 0.7273 |
| MPV | -0.18 | -0.0001 (-0.0013, 0.0012) | 0.000 | 0.9092 |
| Plateletcrit | -2.50 | -0.0005 (-0.0029, 0.0019) | 0.006 | 0.6743 |
| PDW | 0.34 | -0.0001 (-0.0007, 0.0004) | 0.008 | 0.6279 |
| P-LCR | 2.66 | -0.0009 (-0.0044, 0.0026) | 0.009 | 0.6051 |
| ALT | 34.02 | -0.0066 (-0.0132, 0.0001) | 0.116 | 0.0525 |
| AST | 12.34 | -0.0025 (-0.0075, 0.0025) | 0.032 | 0.3168 |
| GGT | -8.09 | 0.0016 (-0.0013, 0.0045) | 0.039 | 0.2720 |
| ALP | 31.95 | -0.0043 (-0.0141, 0.0056) | 0.024 | 0.3873 |
| TC | -4.25 | 0.0005 (-0.0011, 0.0020) | 0.012 | 0.5479 |
| TG | -11.93 | 0.0028 (-0.0018, 0.0075) | 0.047 | 0.2242 |
| HDL | -2.98 | 0.0002 (-0.0014, 0.0019) | 0.003 | 0.7631 |
| LDL | -4.52 | 0.0004 (-0.0012, 0.0020) | 0.007 | 0.6391 |
| UA | -5.49 | 0.0007 (-0.0005, 0.0019) | 0.046 | 0.2300 |
| Urea | -3.45 | 0.0005 (-0.0002, 0.0012) | 0.063 | 0.1588 |
| CREA | -4.70 | 0.0004 (-0.0010, 0.0018) | 0.013 | 0.5296 |
| Glc | -2.83 | 0.0011 (-0.0013, 0.0036) | 0.029 | 0.3462 |

R^2^, the coefficient of determination; Hb, hemoglobin; Hct, hematocrit; MCV, mean cell volume; MCH, mean cell hemoglobin; MCHC, mean cell hemoglobin concentration; RDW-SD, red cell distribution width–standard deviation; Plt, platelet count; MPV, mean platelet volume; PDW, platelet distribution width; P-LCR, Platelet larger cell ratio; ALT, alanine aminotransferase; AST, aspartate aminotransferase; GGT, γ-glutamyltransferase; ALP, alkaline phosphatase; TC, total cholesterol; TG, triglyceride; HDL, high-density lipoprotein cholesterol; LDL, low-density lipoprotein cholesterol; UA, uric acid; CREA, creatinine; Glc, glucose.

**S Table 7. Post-hoc Sensitivity Analysis in Step 2: Minimum Detectable MD at 80% Power *vs*. RCV.**

| **Parameter** | **MDD₈₀, %** | **RCV (95%CI), %** | **≥80% power** |
| --- | --- | --- | --- |
| Leukocytes | 4.35 | (-13.7, 15.9) | Yes |
| Neutrophils | 6.41 | (-16.1, 19.2) | Yes |
| Eosinophils | 10.37 | (-47.7, 91.1) | Yes |
| Basophils | 20.85 | (-57.0, 132.5) | Yes |
| Lymphocytes | 4.73 | (-19.8, 24.6) | Yes |
| Monocytes | 7.46 | (-23.6, 30.9) | Yes |
| Erythrocytes | 1.28 | (-7.8, 8.5) | Yes |
| Hb | 1.33 | (-9.4, 10.4) | Yes |
| Hct | 1.46 | (-7.7, 8.4) | Yes |
| MCV | 0.29 | (-1.6, 1.6) | Yes |
| MCH | 0.46 | (-1.9, 1.9) | Yes |
| MCHC | 0.59 | (-2.4, 2.4) | Yes |
| RDW-SD | 0.62 | (-2.0, 2.1) | Yes |
| Plt | 1.92 | (-13.8, 16.1) | Yes |
| MPV | 1.43 | (-4.9, 5.2) | Yes |
| Plateletcrit | 2.79 | (-12.7, 14.6) | Yes |
| PDW | 0.66 | (-2.1, 2.1) | Yes |
| P-LCR | 4.12 | (-12.9, 14.8) | Yes |
| ALT | 8.22 | (-25.0, 33.2) | Yes |
| AST | 5.95 | (-21.9, 28.1) | Yes |
| GGT | 3.46 | (-17.2, 20.8) | Yes |
| ALP | 11.64 | (-14.8, 17.4) | Yes |
| TC | 1.78 | (-4.6, 4.8) | Yes |
| TG | 5.53 | (-12.9, 14.8) | Yes |
| HDL | 1.93 | (-5.8, 6.2) | Yes |
| LDL | 1.87 | (-5.6, 5.9) | Yes |
| UA | 1.42 | (-6.1, 6.5) | Yes |
| Urea | 0.83 | (-3.8, 3.9) | Yes |
| CREA | 1.62 | (-7.5, 8.2) | Yes |
| Glc | 2.88 | (-16.3, 19.4) | Yes |

MDD₈₀%, minimum detectable MD at 80% power, computed as MDD₈₀% = SE (MD%) × (t₀.₀₂₅, df + t₀.₂₀, df); RCV, reference change value from Step 1; ≥80% Power, checks whether MDD₈₀% ≤ RCV bound; Hb, hemoglobin; Hct, hematocrit; MCV, mean cell volume; MCH, mean cell hemoglobin; MCHC, mean cell hemoglobin concentration; RDW-SD, red cell distribution width–standard deviation; Plt, platelet count; MPV, mean platelet volume; PDW, platelet distribution width; P-LCR, Platelet larger cell ratio; ALT, alanine aminotransferase; AST, aspartate aminotransferase; GGT, γ-glutamyltransferase; ALP, alkaline phosphatase; TC, total cholesterol; TG, triglyceride; HDL, high density lipoprotein cholesterol; LDL, low density lipoprotein cholesterol; UA, uric acid; CREA, creatinine; Glc, glucose.
